# Medical News and Miscellany

**Published:** 1887-09

**Authors:** 


### Medical News and Miscellany.

It is now *Baron* Pasteur, we believe.

The Congress is over, and was a success,—next!

The 10th International Congress will meet in Berlin in 1890.

---

Dr. J. M. Ross, of Belton, lost his residence by fire last month.

---

Dr Dabney, of Kinneyville, was married on the 17th of August, to a young lady in Louisiana.

---

The Climatologist, with Dr. G. H. Rohe as editor, will make its appearance this month, as another Star in the firmament of Medical Journalism. Success to it.

---

Chestnut.—A little girl who had had cocaine injected into the gum for the extraction of a tooth, exclaimed: "U-m-p-h! tastes just like my foot was asleep!"

---

We acknowledge the courtesy of an invitation to dine with Dr. A. R. Robinson of New York, President of the Section on Dermatology, at Willard's in Washington, Sept. 7th, "to meet Foreign and American Dermatologists," and much regret our inability to be present.

---

Wanted: A thoroughly competent and experienced prescription druggist, single man, is open to engagement in some leading drug house. Best of testimonials. Adrress, V. B., care of Editor *Daniel's Texas Medical Journal*, Austin, Texas.

---

"What's the Matter with the Asylums?" It is a singular coincidence that within this month three Superintendents of State Lunatic Asylums have been requested by the Boards of Managers to resign, to-wit: The Superintendent of "Ward's Island Insane Asylum," New York. Dr. W. B. Foster, Superintendent Indiana State Lunatic Asylum, and Dr. J. S. Dorsett, of the Texas State Lunatic Asylum.

An Unjust Slight. Amid all the rejoicing over the success of the Congress there were some sad hearts, sad because of the sting of slight and injustice; and they were those too, who had done most to make it a success. We refer to the General Committee—the planners and organizers of the work. We learn from a reliable source that the Committee did not have, even, honorable mention, but, upon the contrary,—when mentioned—if mentioned at all, a kind of apology was offered or implied, for the selections, about to this effect—"in accordance with our Republican form of Government," — or "in Democratic America ;"—as much as to say the Committee was not what it should have been, and in *more civilized* and enlightened countries, it would have been differently constituted.

This is unjust and even contemptible. The Committee was pre-eminently representative of *American Medicine*, and individually its members were the peers of the ablest *in the world*—and they made sacrifices for the cause, which entitled them to respect. It is shameful, and if intentional, contemptible. We look to see a protest go up from every State—and to hear each Southern and Western Journal speak out in condemnation of it in no uncertain terms.

---

The Alabama Surgical and Gynecological Association extend an invitation to physicians of other States to meet with them at Birmingham on 11th October prox., when it is proposed to organize a South-Western Surgical and Gynecological Association. We acknowledge the courtesy of an invitation to be present, and a special invitation from Dr. Davis, the energetic and polite Secretary, to partake of the hospitalities of his home during the session. We are also asked our opinion "of the feasibility of such organization." The gentlemen have our best wishes for success and we cordially advise physicians who can do so, to attend; meantime to open correspondence with Dr. Davis, who will be pleased to give them all information on the subject.

---

The *American Lancet* refers to our Dallas contemporary as "*The Texas Medical Journal.*" We beg to remind him that this Journal claims the name, and is the only "*Texas Medical Journal.*" There is a difference between a Medical Journal published in Texas, and "*The Texas Medical Journal,*" especially as each has a distinctive title.

DELEGATES CERTIFICATES to the International Medical Congress were issued to the following members T. S. M. A.: R. B. White, Ennis, vice W. B. Brooks, G. B. Hall, Galveston, vice W. G. Jameson, W. Caston, Corsicana, vice F. E. Daniel, L. B. Creath, Kinneyville, vice R. M. Swearingin, R. Rutherford, Houston, State Health Officer, vice F. R. Martin.

---

INDUBITABLE PROOF.—*Lawyer*: "Doctor, did you make an examination of the person of the plaintiff, with reference to this charge of rape?"

*Doctor*: "Yes, sir, yes, sir! made a thorough examination."

*Lawyer*: "Well, Doctor, did you find any evidences that violence had been done her person?"

*Doctor*: "Yes, sir, yes, sir—plenty sign."

*Lawyer*: "Doctor, please state to the court what were those evidences?—what was the condition of the parts, that led you to believe that violence had been done her person?"

*Doctor*, (with a wise look, and telling it off on his fingers ends): "Well, Jedge,—yer see—ther little *connoojum*—what jines on to the *center-piece*, was ON-*t-i-r-e-l-y* sp'ilt!!!"

---

JOURNALISTIC MAVERICS.—In early days in Texas, there was a wealthy ranchman named Maverick. Every spring when branding time came, he would appropriate to himself, and put his brand on every unbranded calf he could find, saying, "that's a maverick." Certain of our exchanges copy certain paragraphs without credit, and get credit themselves, in the next journal that copies it from their pages;—a kind of freebooting that is extensively carried on by some; yet they are ready to kick if it is practiced on them.

About a year ago, we got off a "good thing" on the "Bacillus Decalvans," stating that Prof. Von Schlen had discovered the bacillus of alopecia areata, and had, by inoculation, produced areas of baldness on the lower animals. We expressed the belief that "immortality awaits the man who will discover a microbe that will make the hair grow on bald heads, instead of one that will prevent it." Now, this has gone the rounds, till its parentage is quite forgotten by all but its parents. We have seen it in nearly every journal—without credit; till it finally comes to us in the *St. Louis Weekly Review*, credited to the *Pacific Record*—3000 miles from where it started !

---

STRANGULATED HERNIA.—Dr. W. W. Walker, of Schulenburg, Texas, has a paper on the subject, in the present issue, to which we call especial attention. His experience, for a physician practicing in a small inland town, has been phenomenal, and his success in reduction, equally so. Few country practitioners, or, indeed the more favored physicians of the larger towns, see sixty cases of strangulated hernia, in a life-time. It is the fortune of army surgeons, and those in large hospitals to see many of these cases. In view of the universal and unfailing success of the Doctor's plan of reduction in strangulated inguinal hernia, it would be well for all who are likely to encounter these cases, to make a note of it, and practice it. If the plan succeeds as well in the hands of others, it will hardly be necessary to look any farther,—we have found it. Eureka !

Our own observation has been that, as a rule, taxis is a failure, and an operation is demanded,—and that one reason of the mortality attending the operation is—that it has been postponed too long, the surgeon relying upon a method, which has, at last failed him ; the intestine is either found on the verge of sphacelus, or, a violent inflammation is set up by the handling. Dr. Walker deserves the thanks of the profession for this apparently simple yet successful plan of reduction, whereby an operation, usually very grave and often fatal, may be obviated.

---

FEMININE PHYSICIANS.—A circular was recently sent to members of the Massachusetts Medical Society, asking the following question : "Do you favor the admission of women to the Society on the same terms as men?" Eleven hundred and thirty-two replies were received, of which 700 were affirmative and 400 negative, and 23 being indifferent. The State membership of the Society is 1,343. Answers to other questions show first, that 336 members have consulted with women physicians ; second, that 931 do not object to consulting with women on account of their sex.—*Kansas City Times.*

California is entitled to the credit for being the first State to receive and recognize Lady Physicians, and Texas claims the honor of being the first to receive them into fellowship without a dissenting voice. At the late meeting of the Texas State Medical Association, Dr. Florence E. Collins, an accomplished lady-graduate of Chicago, was received into membership by a rising vote,—the first lady applicant this Association ever had. Dr. Collins is the very efficient Secretary and Treasurer of the Travis County Medical Association.

By the bye, at a late meeting of the Travis County Medical Society, she related the occurence of a most remarkable case coming under her observation :

A REMARKABLE CASE OF URTICARIA.—The patient, a lady of over fifty, had, for more than thirty years, been subject to "attacks" of what appears to be urticaria in an exaggerated form. The eruption occurs suddenly and without prodroma, at about 5 o'clock, p. m., every day, lasts until about 7 o'clock, a. m., next day, and subsides spontaneously, leaving no sequellæ. It is attended with intense itching, and a feeling of distension. The eruption is annular, and appears all over the body; on the trunk, large rolls of thickened skin encircle the body; the same on the neck, and on the limbs, while the lower extremities are œdematous. These attacks occur with as distinct periodicity as chills and fever,—for some three or four months, when they subside, to reappear at intervals of two or three or four years—when they run the same course. No cause can be found for them, and so far, all treatment has been alike unavailing.

We advised quinine, in anti-periodic doses.

---

A DESERVED COMPLIMENT.

At the late Grand Convocation of the Grand Lodge of Knights of Honor of Texas, held in this City, our handsome and talented friend, Dr. Jas. D. Osborn of Cleburne, was unanimously endorsed for re-appointment as State Medical Examiner of the Order. We extend to the Doctor our sincere congratulations.

Apropos to this subject, we are permitted to use the following extract from the minutes of the Grand Secretary, Hon. Wm. P. Cole :

"Concerning the report of our able and efficient State Medical Examiner, James D. Osborn, M. D., the Committee on State of the Order at the recent session of the Grand Lodge K. of H. held in Austin, say; "Upon the report of the State Medical Examiner which was referred to us, your Committee would say, that the report speaks for itself as to the faithfulness and ability with which that exceedingly important part of our work has been carried on, and your Committee think, that the State Medical Examiner is justly entitled to the thanks of the Order for the diligence and ability which he has thrown into his work for us."

The Grand Lodge manifested its high appreciation of the valuable services of Doctor Osborn by unanimously reelecting him to serve for the next ensuing two years."

In placing the Doctor in nomination, Col. T. J. Goree paid a just tribute to a faithful and deserving officer by saying that to the skillful and vigilant watch and management of this officer during the past two years are we indebted more than to all other causes for the great decrease in our mortality.

---

THE following piece of sublime misinformation has been perpetrated by a homœpathic physician of Montreal as a *bona fide* report of a case :

"Called to see Miss U———, suffering from usual periodic headache ; gave *Lach.* 75 m. (Finckle), and then dissolved a few No. 8 pellets of *pure sacch. album* in half a glass of water, *churning it well* with a spoon (potentizing it, I fear); dose, a teaspoonful every half-hour till better......Soon after taking the *sacch. alb.*, she became deadly sick and 'faint ; thought she would *die, she was so weak*, could not lift her head ; *felt as if she would sink bodily right through the bed.*' Each dose made her so much worse that she stopped it, and was able to come down stairs......This is the first time I had such a symptom from *sacch. alb.*, but still it was too marked to pass on without making a note for verification, either clinically or pathogenetically. I know I had pure sugar pellets and fresh water."......

We can't help wondering whether the Examining Board of the Providence of Quebec have applied their final tests to this gentleman, and if so how he stood ; also, whether he could pass the civil service examination here. *Medical Press, N. Y.*

---

DR. EDMUND GOLDMANN.

We regret to learn, that the profession of Texas is soon to loose the companionship of this able and distinguished physician. He will leave Galveston shortly for SAN JOSE, California, where he will reside in future. We commend the Doctor to the profession in his new home, as a gentleman, worthy, in every way of their confidence and esteem, and a physician of a high order of ability, which has won him distinction in his own state. The Doctor is a graduate of the N. O. School of Medicine and the Editor of this JOURNAL claims the distinction of having been a class-mate with him. He is well and favorably known to the medical world by his contributions to the literature of the day,—to this JOURNAL, to the *New York Medical Record*, and other Journals,—his last paper being one on "Mental Alienation the result of Intermittent Fever," read at the Convention of the Texas State Medical Association, and published in the late Volume of Transactions. The Galveston County Medical Society has made the Doctor an honorary member, in anticipation of his departure, he having been, for many years, a zealous active worker in the organization, and it is an open secret that, had he remained, he would have occupied an important chair in the Texas Medical College. *Bon Voyage*, Doctor.

